# Skin Autofluorescence Is Associated with the Progression of Chronic Kidney Disease: A Prospective Observational Study

**DOI:** 10.1371/journal.pone.0083799

**Published:** 2013-12-12

**Authors:** Kenichi Tanaka, Masaaki Nakayama, Makoto Kanno, Hiroshi Kimura, Kimio Watanabe, Yoshihiro Tani, Yuki Kusano, Hodaka Suzuki, Yoshimitsu Hayashi, Koichi Asahi, Keiji Sato, Toshio Miyata, Tsuyoshi Watanabe

**Affiliations:** 1 Departments of Nephrology and Hypertension, Fukushima Medical University, Fukushima, Japan; 2 Department of Chronic Kidney Disease Initiatives, Fukushima Medical University, Fukushima, Japan; 3 Department of Nephrology, Fujita General Hospital, Kunimi, Japan; 4 United Centers for Advanced Research and Translational Medicine, Tohoku University Graduate School of Medicine, Sendai, Japan; University of Florida, United States of America

## Abstract

**Background:**

Advanced glycation end product (AGE) accumulation is thought to be a measure of cumulative metabolic stress that has been reported to independently predict cardiovascular disease in diabetes and renal failure. The aim of this study was to evaluate the association between AGE accumulation, measured as skin autofluorescence, and the progression of renal disease in pre-dialysis patients with chronic kidney disease (CKD).

**Methods:**

Skin autofluorescence was measured noninvasively with an autofluorescence reader at baseline in 449 pre-dialysis patients with CKD. The primary end point was defined as a doubling of serum creatinine and/or need for dialysis.

**Results:**

Thirty-three patients were lost to follow-up. Forty six patients reached the primary end point during the follow-up period (Median 39 months). Kaplan-Meier analysis showed a significantly higher risk of development of the primary end points in patients with skin autofluorescence levels above the optimal cut-off level of 2.31 arbitrary units, derived by receiver operator curve analysis. Cox regression analysis revealed that skin autofluorescence was an independent predictor of the primary end point, even after adjustment for age, gender, smoking history, diabetes, estimated glomerular filtration rate and proteinuria (adjusted hazard ratio 2.58, *P* = 0.004).

**Conclusions:**

Tissue accumulation of AGEs, measured as skin autofluorescence, is a strong and independent predictor of progression of CKD. Skin autofluorescence may be useful for risk stratification in this group of patients; further studies should clarify whether AGE accumulation could be one of the therapeutic targets to improve the prognosis of CKD.

## Introduction

Advanced glycation end products (AGEs), synthesized by the non-enzymatic response of glucose to protein (the Maillard reaction), have been implicated as a contributing factor in the progression of atherosclerosis and cardiovascular disease (CVD) [1], [2], [3]. In addition to hyperglycemia and increased oxidative stress, decrease in glomerular filtration rate (GFR) is thought to be an important determinant contributing to the accumulation of AGEs [4], [5]. AGE accumulation during chronic kidney disease (CKD) may be exacerbated by its increased formation, as oxidative and carbonyl stress are increased with reduced renal function. Accumulation of chemically stable AGEs on long-lived proteins may serve as a measure of cumulative metabolic stress, since these facilitate cellular damage by directly modifying cellular proteins and altering their function or by binding to specific AGE receptors (RAGE) that induce a broad range of cellular responses, leading to injury [6]. While they are implicated as a major pathogenic mechanism in vascular complications in patients with diabetes, AGEs also contribute to kidney injury both in diabetic and non-diabetic renal disease [7]. Therefore, AGEs are thought to be one of the possible biomarkers to assess the development of renal disease in CKD.

An autofluorescence reader (Diagnoptics, Groningen, the Netherlands) non-invasively assesses AGE accumulation using skin autofluorescence under ultraviolet light; skin autofluorescence has been validated against AGE measurements in skin biopsies from the site of skin autofluorescence measurement, performed in healthy controls and patients with diabetes or CKD [8], [9], [10]. Skin autofluorescence is reportedly an independent predictor of cardiovascular mortality in diabetic and hemodialysis patients [9], [11]. We recently reported that skin autofluorescence is independently associated with renal function and CVD history in pre-dialysis patients with CKD [12], and more recently, the relationships of skin autofluorescence to renal and cardiovascular risk factors have been reported in a cross-sectional analysis of patients with CKD stage 3 [13].

However, the prognostic value of skin autofluorescence on the progression of renal disease in pre-dialysis patients with CKD has not been previously reported. Therefore, we hypothesized that increased skin autofluorescence may be one of the clinical risk factors of CKD progression. To test this hypothesis, we investigated whether AGE accumulation, measured as skin autofluorescence, is a predictor of renal outcome in pre-dialysis patients with CKD.

## Methods

### Study population and baseline investigations

We initially examined the baseline characteristics of 449 patients who had CKD and various degrees of renal impairment. These patients were recruited from three nephrology departments in Japan (Fukushima Medical University, Fujita General Hospital, and Tani Hospital). Recruitment was performed between April 2006 and November 2011. The inclusion criteria were as follows: (1) CKD, according to the definition described below, with stable renal function for at least three months before entry into the study, and (2) age 18 years or over. Exclusion criteria were as follows: (1) receiving dialysis treatment in the last three months, (2) active malignancy, (3) infectious disease, (4) pregnancy, and (5) organ transplantation. In addition, 43 patients with skin reflectance below 10% were excluded because of the autofluorescence reader`s limitation of being unable to accurately measure autofluorescence in non-Caucasians with dark skin type [14]. All patients were of Japanese ethnicity. Baseline blood samples from all patients were collected at the clinic by venipuncture in a non-fasting state. Serum creatinine was measured using an enzyme-based method, and low-density lipoprotein (LDL) cholesterol was measured using a direct method. Serum albumin, hemoglobin and high-density lipoprotein (HDL) cholesterol were measured by standardized automated laboratory techniques in the clinical laboratories of each participating institution. Random urine samples were also collected at baseline to measure the ratio of urinary protein to creatinine.

### Ethics statement

The study protocol complied with the Declaration of Helsinki and was approved by the ethics committees of Fukushima Medical University (acceptance no. 762). All patients received an explanation of the procedures and possible risks of this study and provided written informed consent to participate.

### Definitions

Estimated GFR was calculated using the estimation equation for Japanese patients with CKD. This equation calculates GFR from serum creatinine, age and gender using the following formula [estimated GFR (mL/min/1.73 m^2^)  =  194 × Serum creatinine^−1.094^ × Age^−0.287^ (× 0.739 for women)] [15]. CKD was defined as an estimated GFR <60 mL/min/1.73 m^2^ or positive dipstick results for proteinuria (≥1+) [16]. Hypertension was defined as systolic blood pressure of at least 140 mm Hg, diastolic blood pressure of at least 90 mm Hg measured in the clinic on two or more occasions, or a history of treatment with antihypertensive drugs. Dyslipidemia was defined by LDL cholesterol values ≥140 mg/dl, HDL cholesterol values <40 mg/dl, or the use of antidyslipidemic drugs. Diabetes was defined by glucose values ≥200 mg/dL at any time, fasting glucose values ≥126 mg/dL or the use of insulin, glucagon-like peptide-1 analog or oral hypoglycemic drugs.

### Prospective follow-up

The patients were prospectively followed-up until October 2012 or until the study end point was reached. Patients received regular follow-up care in the outpatient ward. The primary kidney end point was defined as a combination of doubling of baseline serum creatinine and end-stage kidney disease requiring kidney replacement therapy. The secondary end point consisted of a composite of the primary kidney end point and all-cause death. Thirty three patients were lost to follow-up. A total of 416 (93%) patients from the baseline cohort could be assessed during the follow-up.

### Skin autofluorescence

Skin AGE levels were assessed based on skin autofluorescence using the AGE Reader, as described previously [8], [10], [17]. Autofluorescence was defined as the average light intensity per nanometer in the range between 420 and 600 nm, divided by the average light intensity per nanometer in the range between 300 and 420 nm. It was expressed in arbitrary units (AU). All measurements were performed at room temperature with the patient in a seated position, at the volar side of the lower arm, approximately 10–15 cm below the elbow fold. The intra- and inter-day assay precision, expressed as coefficients of variation for autofluorescence reader measurements, were 2.5% (*n* = 10) and 4.6% (*n* = 12), respectively. Autofluorescence was calculated offline by automated analysis and was observer-independent.

### Statistical analyses

Statistical analysis was performed using PASW Statistics version 18.0 for windows software. Continuous variables are expressed as median interquartile range (IQR). Discrete data are given as counts and ratios (%). The Mann-Whitney U test and Kruskal-Wallis test were applied to compare differences between groups where appropriate. Spearman’s rank correlation test was used to estimate relationships between variables, and χ^2^ test was used for categorical ones. Multiple linear regression analyses were performed to determine the independent relationships between the variables and skin autofluorescence. Univariate Cox proportional hazards analysis was used to identify the predictors of the study end point. The proportionality of hazard assumption was confirmed graphically. Multivariate Cox proportional hazard models were constructed to assess the independent prognostic roles of skin autofluorescence adjusted for baseline characteristics (age, gender, smoking history, diabetes, hypertension, dyslipidemia, estimated GFR, proteinuria and serum albumin). A receiver operating characteristics curve was drawn and the area under the curve was calculated. The optimal cut-off value of skin autofluorescence was defined as the cut-off value that obtained the highest sum of sensitivity and specificity. Kaplan-Meier curves were generated for patients with skin autofluorescence values above and below the optimal cut-off levels, and the log-rank test was performed to compare skin autofluorescence levels and the incidence of the end point. A two-sided *P*-value <0.05 was considered significant.

## Results

The study was registered in the University Hospital Medical Information Network Clinical Trials Registry (UMIN-CTR) UMIN000012285.

### Clinical and biochemical characteristics

We initially examined the baseline characteristics of 449 CKD patients. Thirty three patients were lost to follow-up, and a total of 416 (93%) patients from the baseline cohort could be assessed during the follow-up. Further analyses were conducted in 416 patients who completed follow-up. Table 1 shows the baseline characteristics of these 416 pre-dialysis patients with CKD according to CKD stage. Patients were stratified into four groups according to CKD stage (stage 1; estimated GFR ≥90, stage 2; estimated GFR 60 to 89, stage 3; estimated GFR 30 to 59, stage 4 and 5; estimated GFR <30 mL/min/1.73 m^2^). A continuous and significant increase in skin autofluorescence was observed across CKD stages. Blood pressure, proteinuria and uric acid increased, and serum albumin, hemoglobin and high-density lipoprotein-cholesterol decreased as CKD stage advanced. The median skin autofluorescence titer was 2.06 AU (IQR, 1.71–2.44 AU, range 0.76–3.90 AU), which negatively correlated with estimated GFR (r = –0.40, *P*<0.001). In addition, skin autofluorescence significantly correlated with age at baseline (r = 0.38, *P*<0.001), hypertension (r = 0.14, *P* = 0.003), dyslipidemia (r = 0.15, *P* = 0.002), diabetes (r = 0.24, *P*<0.001), amount of proteinuria (r = 0.20, *P*<0.001), serum albumin (r = –0.29, *P*<0.001), hemoglobin (r = –0.31, *P*<0.001) and uric acid (r = 0.17, *P*<0.001). Multiple linear regression analysis revealed that 30% (R^2^) of the variance of skin autofluorescence could be predicted by age (β = 0.27, *P*<0.001), diabetes (β = 0.16, *P* = 0.002), estimated GFR (β = –0.14, *P* = 0.03), serum albumin (β = –0.11, *P* = 0.03) and hemoglobin (β = –0.12, *P* = 0.02). Angiotensin-converting enzyme inhibitors (ACEi) or angiotensin II receptor blockers (ARB) were administered to 290 patients (70%).

**Table 1:** Baseline clinical and laboratory characteristics of pre-dialysis patients with chronic kidney disease (CKD) according to CKD stage.

		CKD stage				*p^a^*
Variables	All patients	1	2	3	4 and 5	
*N*	416	36	131	181	68	
Age (y)	64.0 (51.0–74.0)	32.0 (19.3–55.3)	56.0 (46.0–69.0)	69.0 (56.0–77.0)	71.0 (60.3–77.0)	<0.001
Gender, male/female (%)	207/209 (50/50)	20/16 (56/44)	65/66 (50/50)	84/97 (46/54)	38/30 (56/44)	0.5
Body mass index (kg/m^2^)	23.4 (21.3–26.1)	22.2 (20.7–24.4)	23.7 (21.1–26.7)	23.5 (21.5–26.0)	23.4 (21.5–26.0)	0.2
Smoking history, n (%)	138 (33)	9 (25)	39 (30)	64 (35)	26 (38)	0.3
Systolic blood pressure (mm Hg)	133 (120–149)	120 (111–134)	130 (121–145)	134 (120–146)	141 (130–162)	<0.001
Diastolic blood pressure (mm Hg)	77 (69–84)	70 (62–80)	79 (71–88)	76 (68–84)	80 (71–91)	0.001
Serum creatinine (mg/dL)	0.92 (0.75–1.25)	0.64 (0.54–0.77)	0.75 (0.64–0.90)	1.02 (0.87–1.22)	2.90 (2.20–3.85)	<0.001
Estimated GFR (mL/min./1.73 m^2^)	55.8 (41.5–72.1)	96.8 (92.1–110.2)	72.7 (66.8–80.1)	50.1 (43.5–55.3)	15.2 (11.3–22.6)	<0.001
Proteinuria (g/gCr)	0.31 (0.06–1.48)	0.12 (0.03–0.26)	0.33 (0.05–1.02)	0.19 (0.02–1.06)	1.26 (0.50–4.49)	<0.001
Hypertension, n (%)	343 (83)	17 (47)	103 (79)	158 (87)	65 (96)	<0.001
Dyslipidemia, n (%)	240 (58)	10 (28)	74 (56)	108 (60)	48 (71)	<0.001
Diabetes, n (%)	131 (32)	8 (22)	39 (30)	58 (32)	26 (38)	0.4
Skin autofluorescence (AU)	2.06 (1.71–2.44)	1.63 (1.35–2.04)	1.91 (1.58–2.17)	2.17 (1.87–2.52)	2.38 (1.99–2.90)	<0.001
Serum albumin (g/dL)	3.90 (3.50–4.20)	4.10 (3.80–4.30)	4.00 (3.70–4.30)	3.90 (3.60–4.20)	3.50 (2.93–3.90)	<0.001
Hemoglobin (g/dL)	13.0 (11.7–14.2)	14.2 (12.5–14.9)	13.6 (12.8–14.5)	12.9 (11.9–14.1)	10.2 (9.1–11.8)	<0.001
Uric acid (mg/dL)	6.00 (5.00–7.15)	5.05 (4.25–6.18)	5.50 (4.50–6.70)	6.20 (5.30–7.20)	7.20 (6.30–8.00)	<0.001
LDL-cholesterol (mg/dL)	111.0 (90.0–135.0)	99.8 (84.3–113.3)	120.5 (98.0–146.6)	108.0 (87.1–134.1)	106.5 (85.3–137.3)	0.003
HDL-cholesterol (mg/dL)	53.9 (45.0–64.0)	59.5 (46.0–68.7)	57.0 (47.0–68.0)	54.0 (45.3–62.0)	48.0 (35.6–57.0)	<0.001
ARB or ACEi, n (%)	290 (70)	13 (36)	88 (67)	135 (75)	54 (79)	<0.001

*P* ^a^ values were obtained by comparisons across all four groups using the Kruskal-Wallis test and χ^2^ test, as appropriate. CKD stages were determined as follows, stage 1: estimated GFR ≥90, stage 2: estimated GFR 60 to 89, stage 3: estimated GFR 30 to 59, and stage 4 and 5: estimated GFR <30 mL/min/1.73 m^2^, respectively. AU, arbitrary units; LDL, low-density lipoprotein; HDL, high-density lipoprotein; ARB, angiotensin II receptor blocker; ACEi, angiotensin-converting enzyme inhibitor. Data are expressed as median (interquartile range).

### Skin autofluorescence and progression of CKD

The median follow-up time was 39 months (IQR 25–45 months). The primary kidney end point was defined as a combination of doubling of baseline serum creatinine and end-stage kidney disease requiring kidney replacement therapy. The secondary end point consisted of a composite of the primary kidney end point and all-cause death. During the follow-up period, 46 of 416 (11%) patients reached the primary kidney end point (18 patients showed doubling of serum creatinine and 28 patients progressed to end-stage kidney disease requiring dialysis). In addition, 25 of 416 (6%) patients died during the follow-up period (16 patients died before reaching the primary kidney end point), and 62 patients reached the secondary end point. The causes of death were as follows: cardiac failure (seven cases); malignancy (five cases); sepsis (three cases); gastrointestinal hemorrhage (two cases); end-stage kidney disease (two cases); arrhythmia (one case); respiratory failure (one case); accident (one case); senility (one case); sudden death (one case); and unknown (one case). Patients whose disease progressed to the primary kidney end point were significantly older and had lower estimated GFR and higher proteinuria (Table 2). In addition, they had significantly higher skin autofluorescence and lower serum albumin and hemoglobin. The level of skin autofluorescence was also significantly higher in patients who reached the secondary end point than in those who did not [median (IQR) 2.54 AU (2.11–2.99) versus 1.99 AU (1.66–2.33), *P*<0.001].

**Table 2:** Comparison of baseline characteristics between patients who did and did not reach the primary kidney end points.

Variables	End point (−)	End point (+)	*p^a^*
*N*	370	46	
Age (y)	62.0 (49.0–73.0)	70.5 (57.8–78.0)	0.004
Gender, male/female (%)	185/185 (50/50)	24/22 (48/52)	0.8
Body mass index (kg/m^2^)	23.3 (21.3–26.2)	23.6 (21.4–25.7)	0.7
Smoking history, n (%)	124 (34)	14 (30)	0.4
Systolic blood pressure (mm Hg)	132 (120–146)	139 (126–162)	0.02
Diastolic blood pressure (mm Hg)	77 (69–84)	79 (66–90)	0.9
Serum creatinine (mg/dL)	0.90 (0.74–1.15)	2.43 (1.09–3.70)	<0.001
Estimated GFR (mL/min./1.73 m^2^)	58.1 (46.1–73.6)	19.1 (12.0–48.4)	<0.001
Proteinuria (g/gCr)	0.24 (0.04–1.04)	4.33 (1.93–8.68)	<0.001
Hypertension, n (%)	300 (81)	43 (94)	0.04
Dyslipidemia, n (%)	207 (56)	33 (72)	0.04
Diabetes, n (%)	106 (29)	25 (54)	<0.001
Skin autofluorescence (AU)	2.00 (1.66–2.36)	2.64 (2.21–3.05)	<0.001
Serum albumin (g/dL)	3.90 (3.60–4.20)	3.35 (2.65–3.90)	<0.001
Hemoglobin (g/dL)	13.2 (12.0–14.3)	10.5 (9.3–12.0)	<0.001
Uric acid (mg/dL)	5.80 (4.90–7.00)	7.10 (6.28–8.30)	<0.001
LDL-cholesterol (mg/dL)	111.0 (91.4–134.6)	112.5 (81.8–141.0)	0.9
HDL-cholesterol (mg/dL)	54.0 (46.0–64.0)	49.6 (35.9–59.0)	0.02
ARB or ACE inhibitor, n (%)	251 (68)	39 (85)	0.02

*P* ^a^ values were obtained by comparison across the 2 groups using the Mann-Whitney test and χ^2^ test, as appropriate. AU, arbitrary units; LDL, low-density lipoprotein; HDL, high-density lipoprotein; ARB, angiotensin II receptor blocker; ACEi, angiotensin-converting enzyme inhibitor. Data are expressed as median (interquartile range).

**Figure 1:**
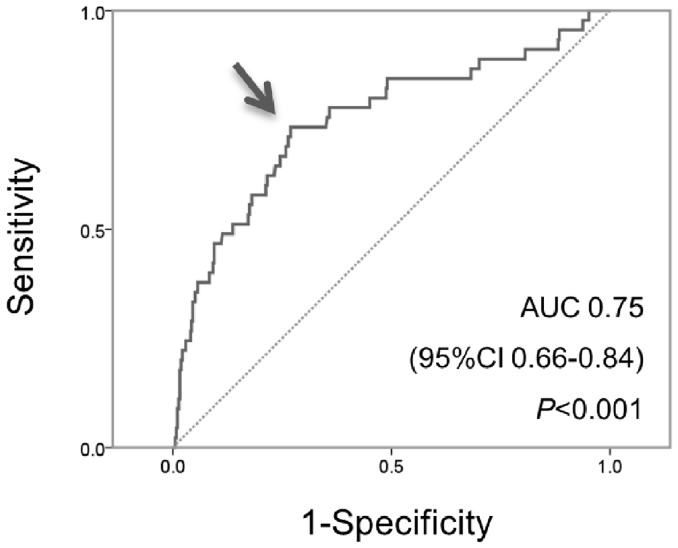
Receiver operating characteristics curve for skin autofluorescence to determine the kidney end point. The area under the curve (AUC) for skin autofluorescence was 0.75, and skin autofluorescence level of 2.31 AU was the optimal cut-off value for the primary kidney end point (sensitivity 0.74, specificity 0.73). The optimal cut-off value of skin autofluorescence was defined as the cut-off value that obtained the highest sum of sensitivity and specificity.

**Figure 2:**
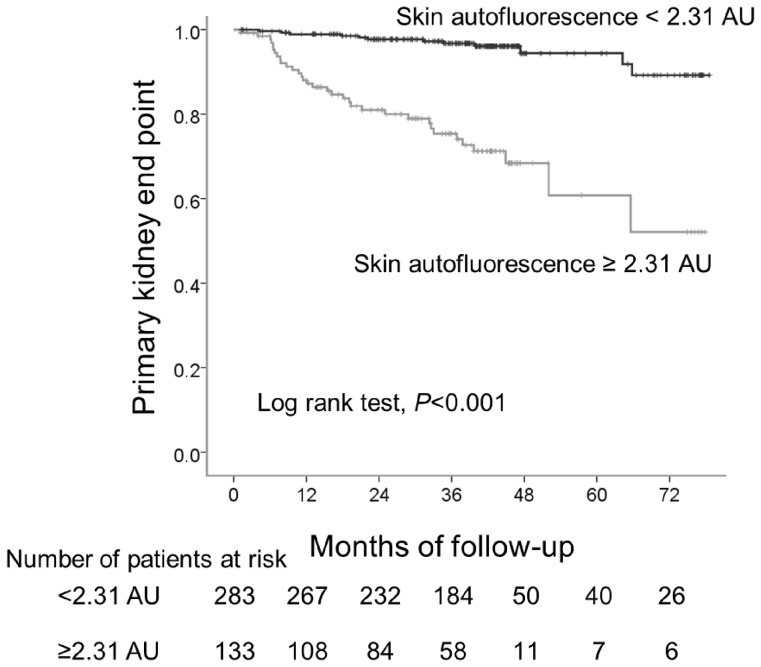
The primary kidney end point-free survival curve. Compared with patients with skin autofluorescence levels below the optimal cut off value (2.31 AU), kidney end point-free survival was significantly lower in patients with skin autofluorescence level above 2.31 AU (*P*<0.001).

**Figure 3:**
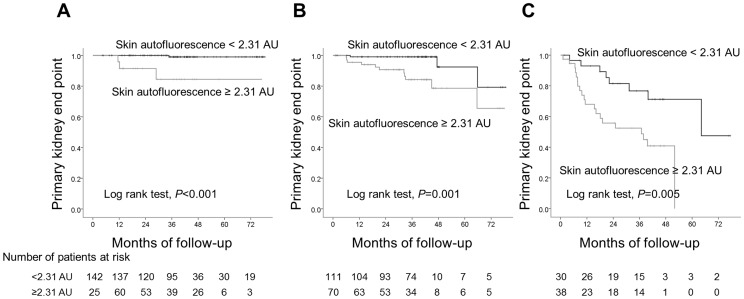
The primary kidney end point-free survival curve. Patients with CKD stage 1+2 (A), CKD stage 3 (B), and stage 4+5 (C).

Receiver operating characteristics analysis revealed that a skin autofluorescence level of 2.31 AU was the optimal cut-off value for the primary kidney end point [area under curve 0.75, 95% confidence interval (CI) 0.66–0.84, *P*<0.001, sensitivity 0.74, specificity 0.73] (Figure 1). Kaplan-Meier curve analysis revealed that patients with autofluorescence above the cut-off level (2.31 AU) reached the primary kidney end point more frequently than those below this level (Figure 2). The results of univariate Cox regression analysis indicated that skin autofluorescence was a predictor of development of the primary kidney end point, together with diabetes, dyslipidemia, estimated GFR, amount of proteinuria and serum albumin (Table 3). Age- and gender-adjusted Cox regression analysis revealed that estimated GFR, amount of proteinuria, serum albumin and skin autofluorescence showed strong associations with the primary kidney end point (Table 3, model 1). After adjustment for baseline covariates, skin autofluorescence remained a significant predictor of progression of CKD (Table 3, model 2). Diabetes, estimated GFR and proteinuria showed borderline significance as predictors of progression, and serum albumin was not independently associated with CKD progression. Skin autofluorescence also had a significant predictive value for development of the secondary end point based on univariate Cox regression analysis (hazard ratio (HR), 3.76, 95% CI, 2.58–5.48, *P*<0.001), the predictive value remaining significant after adjustment for the baseline covariates is displayed in Table 3 (adjusted HR, 2.05, 95% CI, 1.17–5.60, *P* = 0.01). Sub-analysis performed in patients with CKD stage 1+2, stage 3, and stage 4+5 revealed that skin autofluorescence was significantly related to progression to the primary kidney end point in each stage (Figures 3A, 3B, and 3C, respectively). Age- and gender-adjusted Cox regression analysis revealed significant associations between skin autofluorescence and the primary kidney end point at each stage of CKD (stage 1+2: adjusted HR 10.70, 95% CI, 2.07–55.35, *P* = 0.005; stage 3: adjusted HR, 6.63, 95% CI, 2.42–18.2, *P*<0.001; stage 4+5: adjusted HR, 1.88, 95% CI, 1.05–3.35, *P* = 0.03).

**Table 3:** Cox proportional hazard analysis of predictability of the risk of disease progression to the primary kidney end points.

Variables	Univariate			Model 1			Model 2		
	HR	95%CI	*P*	HR	95%CI	*P*	HR	95%CI	*P*
Smoking history	1.24	0.595–2.564	0.6	2.03	0.786–5.235	0.1	1.25	0.407–3.838	0.7
Diabetes	2.48	1.374–4.488	0.003	2.09	1.137–3.824	0.02	2.40	1.031–5.576	0.04
Hypertension	2.50	0.774–8.096	0.1	1.86	0.562–6.124	0.3	1.54	0.319–7.419	0.6
Dyslipidemia	2.05	1.073–3.910	0.03	1.84	0.960–3.513	0.07	1.07	0.432–2.667	0.9
Estimated GFR (mL/min./1.73 m^2^)	0.94	0.926–0.956	<0.001	0.94	0.926–0.956	<0.001	0.98	0.958–0.998	0.03
Proteinuria (g/gCr)	1.16	1.109–1.209	<0.001	1.15	1.097–1.201	<0.001	1.11	1.014–1.215	0.02
Serum albumin (g/dL)	0.45	0.348–0.591	<0.001	0.44	0.330–0.579	<0.001	0.75	0.418–1.357	0.4
Skin autofluorescence (AU)	4.43	2.867–6.833	<0.001	4.26	2.639–6.878	<0.001	2.58	1.342–4.969	0.004

Model 1, adjusted for age and gender; Model 2, adjusted for all variables in this table; HR, hazard ratio; CI, confidence interval; AU, arbitrary units.

**Figure 4:**
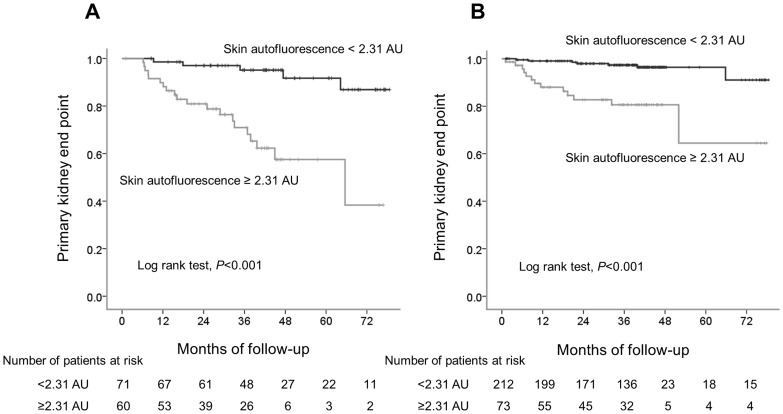
The primary kidney end point-free survival curve. Patients with diabetes (A), and patients without diabetes (B).

Associations between skin autofluorescence and renal outcome in patients with and without diabetes were also investigated; skin autofluorescence was found to have significant associations with the primary kidney end point in both diabetic and non-diabetic patients with CKD by univariate Cox regression analysis (HR, 4.85; 95%CI, 2.58–9.11; *P*<0.001; and HR, 3.75; 95% CI, 1.96–7.17; *P*<0.001, respectively), and that these associations remained significant even after adjustment for age and gender (adjusted HR, 5.97; 95% CI, 3.07–11.62; *P*<0.001; and adjusted HR, 2.64; 95% CI, 1.25–5.57; *P* = 0.01, respectively). Kaplan-Meier curve analysis showed that skin autofluorescence was significantly related to the primary kidney end point in both groups of CKD patients (Figures 4A and 4B).

## Discussion

The results of this prospective study in a sizable cohort of pre-dialysis patients with CKD indicate that skin autofluorescence is a strong and independent predictor of CKD progression. Our results confirm the clinical correlation of CKD progression with recognized risk factors, such as diabetes, baseline renal function and proteinuria [18], [19]. Moreover, skin autofluorescence was significantly related to renal prognosis at each stage of CKD. Thus, the present study shows the predictive value of AGE accumulation, measured as skin autofluorescence, for the renal outcome of CKD. Skin autofluorescence measurement may therefore represent a useful method in daily practice for assessing the renal risk in CKD patients.

The role of AGE accumulation in disease progression, which has been mainly studied in patients with diabetes, has shown that AGE accumulation is one of the contributing factors in the progression of diabetic vascular complications [20], [21]. Skin autofluorescence reportedly has a strong predictive value for the progression of chronic vascular complications and CVD in diabetes [11], [22]; recently, we reported that skin autofluorescence had an independent relationship to vascular complications, such as diabetic retinopathy and nephropathy, in Japanese patients with diabetes [23]. However, AGEs are significantly increased in patients with diminished renal function, even in the absence of hyperglycemia, and also contribute to the progression of kidney disease in non-diabetic nephropathy, the possible mechanisms for this including binding to RAGE, inducing oxidative stress, endothelial dysfunction, inflammation and podocyte injury [7], [24], [25]. Therefore, a sub-analysis of patients with and without diabetes was performed. It was demonstrated that skin autofluorescence exhibited independent predictive value in the progression of renal disease in both diabetic and non-diabetic groups, even after adjusting for age and gender. Skin autofluorescence could thus be useful as a clinical biomarker for renal risk assessment in patients without diabetes.

The independent determinants of skin autofluorescence in this study were age, diabetes, estimated GFR, proteinuria, serum albumin and hemoglobin. All of these determinants have been recognized as risk factors for the progression of kidney disease and/or CVD by previous investigations. AGE accumulation may be closely related to the non-traditional risk factors for CKD progression and CVD, such as reduced GFR, malnutrition, anemia and oxidative stress, as well as aging and hyperglycemia, and represent a complex of these risk factors. Moreover, the power of skin autofluorescence as a prognostic factor for CKD progression is illustrated by the fact that it was found to fare better in Cox regression analysis than the prognostic values of the other known risk factors.

The present study did have some limitations. First, the current autofluorescence reader is not reliable for individuals with very dark skin, as measurement of autofluorescence may be affected by skin color and pigmentation due to the high absorption grade of excited light [10], [26], [27]. Since all patients in the present study were of Japanese ethnicity, patients with skin reflectance below 10% were excluded from the study in accordance with a recent report that the threshold value of skin reflectance (R%) below which skin autofluorescence becomes unreliable is R% = 10% in a non-Caucasian (Chinese) population [14]. The values of skin autofluorescence in our patients were lower than those in other previous studies. The values of skin autofluorescence in patients with CKD stage 3 were 2.2 AU in the present study and 2.7 AU in the study reported by McIntyre NJ et al, which included 1707 patients with CKD stage 3, although the mean age of patients in this study was higher than our patients with CKD stage 3 (72.9 y vs. 69.0 y) [13]. Our results might suggest the racial differences of AGE accumulation between Caucasian and non-Caucasian patients, and this might be due to high absorption grade of both excitation and emission light with darker skin type. Several recent studies have reported skin autofluorescence results in Japanese patients with various diseases, including rheumatoid arthritis [28], cerebral infarction [29], and end-stage kidney disease [30], [31], [32], and demonstrated its potential to be a useful marker in non-Caucasian subjects. However, difference of reference values between races is rather important in clinical practice, therefore, this problem need more investigation and is still open to discuss. The manufacturers of the autofluorescence reader have announced that they are developing improvements to the software that will allow skin autofluorescence measurements even in individuals with darker skin. Second, as this was an observational study, whether reduction of the skin autofluorescence level would result in better renal prognosis remains unclear. Several studies have reported that some AGE breakers reduce accumulation of AGEs by several mechanisms, which include cleaving pre-formed AGE cross-links, reducing accumulation of carbohydrate as well as lipid derived AGEs, and potent free radical scavenging activity, thus inhibiting the development of vascular disease in experimental animals [33], [34], [35]. Further studies are needed to determine whether treatment to reduce AGE accumulation will result in improved renal outcomes. Skin autofluorescence levels have the potential to be important surrogate markers to monitor the effects of these treatments when these drugs are applied in clinical practice. As assessment of glucose at fasting state or at any time might not have covered all patients, definition of diabetes could be one of the limitations to the present study.

In the present prospective study comprising a cohort of pre-dialysis patients with CKD, AGE accumulation, measured as skin autofluorescence, was identified as a novel predictor of CKD progression. The present study is the first to show the prognostic power of AGE accumulation for renal outcome in CKD. Thus, the non-invasive and convenient autofluorescence readers may provide useful biomarkers for risk stratification and detection of high risk cases in early stages of CKD, potentially improving patient outcome.
